# Disseminated cryptococcosis manifested as a single tumor in an immunocompetent patient, similar to the cutaneous primary forms*

**DOI:** 10.1590/abd1806-4841.20164582

**Published:** 2016

**Authors:** Danielle Mechereffe do Amaral, Ritha de Cássia Capelato Rocha, Luiz Euribel Prestes Carneiro, Dewton Moraes Vasconcelos, Marilda Aparecida Milanez Morgado de Abreu

**Affiliations:** 1 Universidade do Oeste Paulista (Unoeste) - Presidente Prudente (SP), Brazil; 2 Universidade de São Paulo (USP) - São Paulo (SP), Brazil

**Keywords:** Cryptococcosis, Host-pathogen interactions, Immunity, Meningitis, cryptococcal

## Abstract

Cryptococcosis is a fungal infection caused by *Cryptococcus neoformans* that tends to affect immunocompromised individuals. The fungi are mostly acquired by inhalation, which leads to an initial pulmonary infection. Later, other organs - such as the central nervous system and the skin - can be affected by hematogenous spread. In addition, cutaneous contamination can occur by primary inoculation after injuries (primary cutaneous cryptococcosis), whose diagnosis is defined based on the absence of systemic involvement. The clinical presentation of cutaneous forms typically vary according to the infection mode. We report an unusual case of disseminated cryptococcosis in an immunocompetent patient with cutaneous lesions similar to those caused by primary inoculation. This clinical picture leads us to question the definition of primary cutaneous cryptococcosis established in the literature.

## INTRODUCTION

Cryptococcosis is a fungal infection caused by two varieties of *Cryptococcus neoformans,* which are divided into five serotypes: serotypes A, D, and AD* (C. neoformans var. neoformans) and* serotypes B and C* (C. neoformans var. gatti)*.^1^

Serotype A has a worldwide distribution and is found in most of Brazil; serotype D is found mainly in the Europe; and *C. neoformans var. gatti*i is limited to tropical and subtropical areas.^2^

This fungus has a wide environmental distribution and is found in tree branches, bird feces, soil, fruit, and vegetables.^3^ Low global frequency of cryptococcosis indicates that the host immune response is sufficient to prevent demonstrations in most immunocompetent patients. However, the disease has been increasingly found in immunocompromised patients, especially HIV-infected individuals.^1,2^

The disease is spread mostly through inhalation of fungi, which usually cause asymptomatic pulmonary infection. However, the disease can spread through the blood, affecting preferably the central nervous system (CNS) and skin, called disseminated cryptococcosis (DC). Another portal of entry is the inoculation of the fungus via skin injury (primary cutaneous cryptococcosis - PCC).^4^

To be considered a primary cutaneous disorder, the disease must have the following features: be exclusively cutaneous, have positive culture for fungus, and show absence of systemic involvement.^5^

Cryptococcosis diagnosis is made by histopathology of the infected tissue, direct detection of fungus in body fluids with India ink examination, isolation in tissue culture, and detection of cryptococcal polysaccharide antigen in the serum and in the cerebrospinal fluid through agglutination by latex or ELISA. The latter test has a prognostic significance (high titers can be predictors of disseminated disease) and serves for therapeutic monitoring.^6^

If infection is present in one area, without CNS involvement or immunosuppression, the treatment is conducted with oral fluconazole, 400 mg/daily for 6-12 months. For cryptococcal meningitis, recommended treatment consists of a two-week induction therapy with intravenous amphotericin B, 0.7-1 mg/kg/day, followed by a consolidation phase with oral fluconazole 800mg/day for eight weeks. Thereafter, maintenance therapy with fluconazole 400 mg/day is suggested for 6-12 months, or until immune restoration.^7^

We report an unusual case of DC in an immunocompetent patient with skin lesions similar to those caused by primary cutaneous inoculation, which leads us to question the accepted definition of PCC and DC as distinct entities.

## CASE REPORT

We report a 68-year-old white male patient born and raised in Tupi (SP), rural worker, who complained about a 30-day asymptomatic lesion on his right forearm, with progressive growth and subsequent ulceration. He reported that he had handled a mortar bag two days before the lesion occurred.

**Figure 1:**
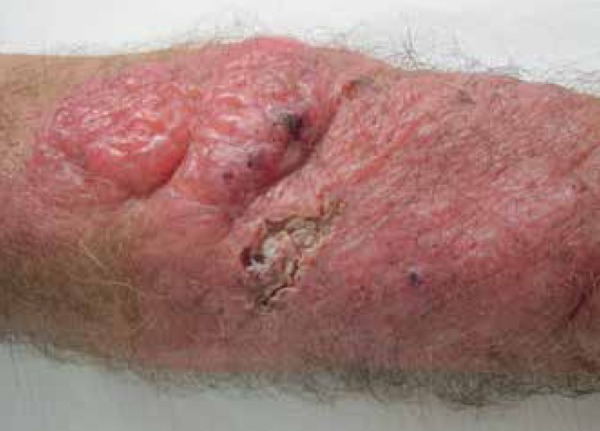
Erythematous plaque with multiple soft and friable nodules

**Figure 2:**
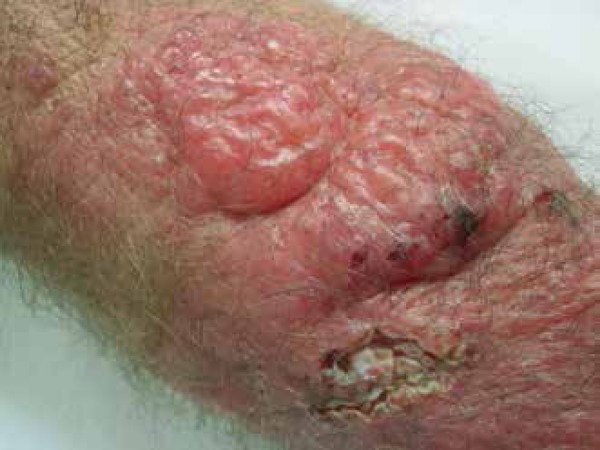
Details of the nodules

**Figure 3:**
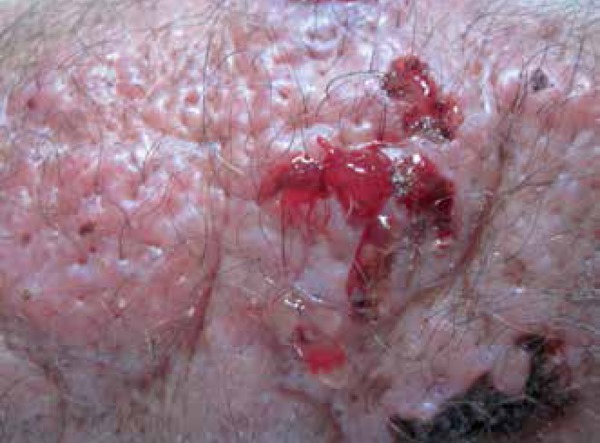
Gelatinous secretion

The patient presented with poorly defined erythematous plaque (about 25cm in size) on the right forearm, displaying multiple nodules on the surface, some with fluctuation and discharging gelatinous secretion after puncture, and some ulcerated areas covered by crusts (Figures 1-3).

He also reported holocranial tension headache, of low intensity, three days before, associated with nausea and asthenia. He denied fever.

The patient revealed a personal history of chronic obstructive pulmonary disease for 10 years, with chronic use of corticosteroid inhalers. He was a smoker (100 packs/year) and ex-alcoholic.

## General physical examination was normal.

**Figure 4:**
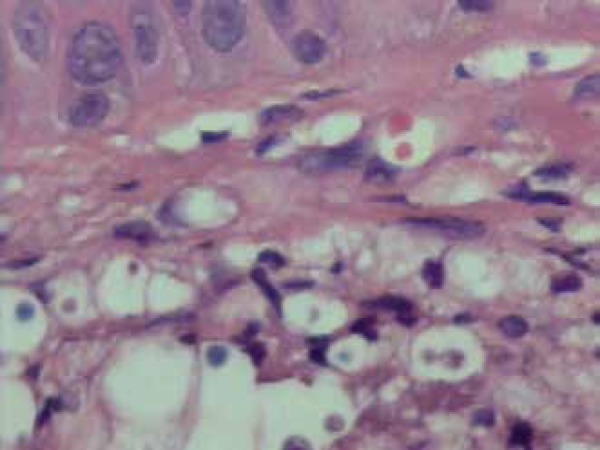
Histopathological examination (HE): encapsulated yeast in the dermis

**Figure 5:**
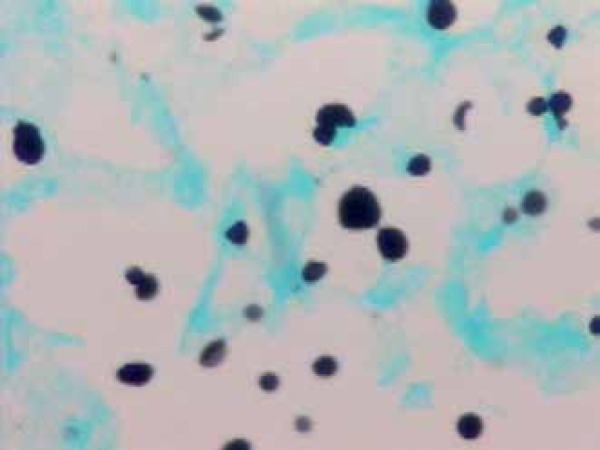
Grocott-Gomori-stained budding yeasts

Histopathological examination of the skin biopsy showed granulomas in the dermis with presence of intermingled fungi morphologically compatible with *Cryptococcus spp* (Figure 4). Mucicarmine and Grocott-Gomori staining showed numerous yeasts in budding (Figure 5).

Culture for fungi revealed brownish yeast colony with creamy and viscous appearance, whose microcultivation showed encapsulated budding blastoconidia, features of *Cryptococcus neoformans*. Cultures for bacteria and mycobacteria were negative.

In systemic investigation, computed tomography was consistent with pulmonary emphysema, with no other relevant findings; CT scans of the abdomen and skull were normal. Fungal blood and urine cultures were negative. Examination of the cerebrospinal fluid showed lymphocytic meningitis attributed to cryptococcosis. Cryptococcal antigen survey in CSF was positive (1:1024).

Based on the findings, we diagnosed DC with proven skin and neurological involvement.

Serology for HIV, serum immunoglobulins (IgG, IgM, and IgA), protein electrophoresis, blood count, biochemical tests, and immunophenotyping of peripheral blood (CD4, CD8, CD19, CD56, CD45RO/CD4) were normal.

Our patient was treated with intravenous liposomal amphotericin B, 5mg/kg/day for two weeks, followed by fluconazole, with a 12-month treatment. Response to treatment was favorable showing an almost complete remission of cutaneous lesions in six months. The patient is being followed up.

## DISCUSSION

Skin involvement in DC patients occurs in approximately 10% of cases, which may be the primary manifestation of the disease and an early sign of dissemination.^2,4,8^ However, PCC is rare and still controversial.

PCC occurs in a population with different personal features (age, gender), immune status, geographic locations compared to DC, suggesting that the diseases are clinically distinct entities. PCC has a predilection for immunocompetent or immunocompromised elderly male patients residents of rural areas.^9^ Cellulitis and ulceration in a limited and exposed cutaneous area, especially on the upper limbs, are the most common manifestations. Gelatinous exudation is common. Most cases present a local trauma history and exposure to bird feces.^3^

DC cutaneous lesions usually spread affecting unexposed and exposed areas. Lesions can appear in any type: umbilicated (mimicking molluscum contagiosum), acneiform, nodular, herpetiform, and cellulitis-like lesions.^4,9^ However, lesions mimicking PCC, as reported in our case, are an exceptional feature.

Systemic cryptococcal infection in immunocompetent patients, as in our case, is rare. Some cases of opportunistic infections have been reported in chronic users of high doses of inhaled corticosteroids, particularly pulmonary aspergillosis, but no DC report was found in the literature.^10^ Despite the prolonged use of inhaled corticosteroids by our patient, we observed no immunosuppression in the immunologic research.

Based on our report, we question, therefore, the validity of the current concept of PCC and DC as different entities. Our patient lived in the rural area, was apparently immunocompetent, and presented with a lesion on an exposed area, features suggestive of PCC. However, the presence of meningitis defined the diagnosis of DC. The results point to the need for comprehensive systemic investigation in all cases of cryptococcosis, even in immunocompetent patients with localized cutaneous lesions. Non-diagnosis of occult systemic alterations can result in catastrophic consequences to the patient due to inappropriate treatment.

## References

[1] Nasser N, Nasser N, Vieira AG (2011). Criptococose cutânea primária em paciente
imunocompetente. An Bras Dermatol.

[2] Spiliopoulou A, Anastassiou ED, Christofidou M (2012). Primary cutaneous cryptococcosis in immunocompetent
hosts. Mycoses.

[3] Leão CA, Ferreira-Paim K, Andrade-Silva L, Mora DJ, da Silva PR, Machado AS (2011). Primary cutaneous cryptococcosis caused by Cryptococcus gattii in
an immunocompetent host. Med Mycol.

[4] Tabassum S, Rahman A, Herekar F, Masood S (2013). Cryptococcal meningitis with secondary cutaneous involvement in
an immunocompetent host. J Infect Dev Ctries.

[5] Marques SA, Bastazini I, Martins AL, Barreto JA, Barbieri D'Elia MP, Lastória JC (2012). Primary cutaneous criptococcosis in Brazil: report of 11 cases in
immunocompetent and imunossupressed patients. Int J Dermatol.

[6] Perfect JR, Bicanic T (2015). Cryptococcosis diagnosis and a treatment: What do we know
now. Fungal Genet Biol.

[7] Perfect JR, Dismukes WE, Dromer F, Goldman DL, Graybill JR, Hamill RJ (2010). Clinical practice guidelines for the management of cryptococcal
disease: 2010 update by the Infectious Disease Society of
America. Clin Infect Dis.

[8] Pau M, Lallai C, Aste N, Aste N, Atzori L (2010). Primary cutaneous cryptococcosis in an immunocompetent
host. Mycoses.

[9] Neuville S, Dromer F, Morin O, Dupont B, Ronin O, Lortholary O (2003). Primary cutaneous cryptococcosis: a distinct clinical
entity. Clin Infect Dis.

[10] Peter E, Bakri F, Ball DM, Cheney RT, Segal BH (2002). Invasive pulmonar filamentous fungal infection in a patient
receiving inhaled corticosteroid therapy. Clin Infect Dis.

